# An Intervention Delivered by App Instant Messaging to Increase Acceptability and Use of Effective Contraception Among Young Women in Bolivia: Protocol of a Randomized Controlled Trial

**DOI:** 10.2196/resprot.8679

**Published:** 2017-12-18

**Authors:** Ona L McCarthy, Veronica Osorio Calderon, Shelly Makleff, Silvia Huaynoca, Baptiste Leurent, Phil Edwards, Jhonny Lopez Gallardo, Caroline Free

**Affiliations:** ^1^ The London School of Hygiene and Tropical Medicine London United Kingdom; ^2^ Centro de Investigación, Educación y Servicios – Salud Sexual Salud Reproductiva La Paz Bolivia; ^3^ International Planned Parenthood Federation/Western Hemisphere Region New York, NY United States

**Keywords:** behavior change, Bolivia, young adult, adolescent, contraception behavior, smartphone, cell phone, reproductive health

## Abstract

**Background:**

Unintended pregnancy is associated with numerous poorer health outcomes for both women and their children. Fulfilling unmet need for contraception is essential in avoiding unintended pregnancies, yet millions of women in low- and middle-income countries continue to face obstacles in realizing their fertility desires. In Bolivia, family planning progress has improved in recent decades but lags behind other countries in the region. Unmet need for contraception among women aged 15 to 19 years is estimated to be 38%, with the adolescent fertility rate at 70 per 1000 women. Mobile phones are an established and popular mode in which to deliver health behavior support. The London School of Hygiene & Tropical Medicine and the Centro de Investigación, Educación y Servicios in Bolivia have partnered to develop and evaluate a contraceptive behavioral intervention for Bolivian young women delivered by mobile phone. The intervention was developed guided by behavioral science and consists of short instant messages sent through an app over 4 months.

**Objective:**

The objective of this study is to evaluate the effect of the intervention on young women’s use of and attitudes toward the most effective contraceptive methods.

**Methods:**

We will allocate 1310 women aged 16 to 24 years with an unmet need for contraception in a 1:1 ratio to receive the intervention messages or the control messages about trial participation. The messages are sent through the Tú decides app, which contains standard family planning information. Coprimary outcomes are use and acceptability of at least one effective contraceptive method, both measured at 4 months.

**Results:**

Recruitment commenced on March 1, 2017 and was completed on July 29, 2017. We estimate that the follow-up period will end in January 2018.

**Conclusions:**

This trial will evaluate the effect of the intervention on young women’s use of and attitudes toward the (nonpermanent) effective contraception methods available in Bolivia.

**Trial Registration:**

ClinicalTrials.gov NCT02905526; https://clinicaltrials.gov/ct2/show/NCT02905526 (Archived by WebCite at http://www.webcitation.org/6vT0yIFfN)

## Introduction

The desire to limit and space childbirth has increased in recent decades, yet many women continue to face obstacles in avoiding unintended pregnancies [1]. Unintended pregnancy is associated with numerous poorer health outcomes for both women and their children [2]. Women with unintended pregnancies are more likely to experience depression and anxiety [3-11], and to initiate prenatal care later [5,11-15] and less frequently [5,12,15]. Unintended pregnancies also increase the risk of low birth weight and preterm birth [16,17]. With young women in particular, unintended pregnancy can delay or prevent educational and career achievements, which can affect future financial security [2]. Where safe abortion is restricted, unintended pregnancies can increase the occurrence of unsafe abortions [18,19]. Satisfying unmet need for contraception is essential in avoiding unintended pregnancy, which requires an understanding of the reasons for nonuse in particular contexts [20].

Bolivia is classified as a lower middle-income country. While the country has experienced recent economic growth, in 2015 around 39% of people were living below the national poverty line [21]. Income inequality is high [21], with substantial inequality between indigenous and nonindigenous populations [22]. Compared with other countries in the region, in Bolivia, progress in family planning has lagged behind [23]. Effective contraception methods are those with a less than 10% typical-use failure rate at 12 months [24-26]; the (nonpermanent) effective methods available in Bolivia are oral contraceptive pills, intrauterine devices, injectables, implants, and the patch. Despite the availability of these methods, the 2008 Bolivian Demographic and Health Survey estimated unmet need among women aged 15 to 19 years to be 38% [27,28]. World Bank indicators for 2015 report the adolescent fertility rate to be 70 per 1000 women aged 15 to 19 years [21]. Abortion is illegal in Bolivia except in cases of rape, incest, and danger to the health of the woman [29]. While there are no official figures on induced abortion, research suggests that around 100 illegal abortions are carried out per day [27], the majority of which are likely to be unsafe due to the legal restrictions on abortion in the country. A survey in 2008 found that, among unmarried sexually active women aged 15 to 19 years, 84% reported wanting to avoid a pregnancy in the next 2 years, but only 49% reported using any contraceptive method [28]. The main reasons given for not using contraception were not being married (52%) (sex before marriage is stigmatized in Bolivia) or having infrequent sex (55%) [28].

Mobile phones are now an established and popular mode in which to deliver health interventions [30-41]. An advantage of using mobile phones to deliver health support is that content can be received at a time of the recipient’s choosing, which may be particularly important with sensitive topics such as sexual and reproductive health. Mobile phone interventions can be delivered through a variety of ways: through voice messages, text messages, mobile apps, instant messages that include videos and images, or bidirectional teleconsultation with health care professionals via text message or a live voice call, to name just a few. While there is some evidence from high-income countries that mobile phone-based interventions can increase contraceptive use [42-44] and knowledge [45], none of the trials evaluating these interventions had a low risk of bias [46]. To the best of our knowledge, only 1 trial has been conducted in a nonhigh-income country (Cambodia); this trial found that postabortion voice messaging with telephone counselling support increased effective contraceptive use at 4 months [47]. Since 2007, mobile phone ownership in Bolivia has increased sharply, with 92 mobile phone subscriptions per 100 people in 2015 [48], which is likely to be higher among younger people.

The London School of Hygiene & Tropical Medicine (LSHTM) and the Centro de Investigación, Educación y Servicios (CIES), a Member Association of the International Planned Parenthood Federation (IPPF), are collaborating to evaluate a contraception intervention delivered by mobile phone for young women in Bolivia. The intervention is informed by integrated behavioral model [49], consists of short mobile phone app instant messages, and is delivered over 4 months through CIES’s Tú decides app. The intervention messages provide accurate information about contraception and include 10 behavior change methods [50]. It was developed through collaboration between LSHTM, CIES, and young people in La Paz and El Alto, Bolivia, with the support of the IPPF Western Hemisphere Region. The collaboration involved various activities aimed at understanding young people’s knowledge of, attitudes toward, and barriers faced in using contraception and preferences for intervention delivery. Guided by behavioral science [51], the intervention was produced through an iterative process of writing, testing with the target group, and refining.

We present the protocol for the evaluation of the intervention by randomized controlled trial. The aim of the trial is to establish whether the intervention increases young Bolivian women’s use and acceptability of effective contraceptive methods.

## Methods

### Study Design

This study is a parallel-group, individually randomized superiority trial with a 1:1 allocation ratio evaluating the effect of an intervention delivered by mobile app. Participants randomly allocated to the intervention arm will have access to the app and will receive the intervention instant messages. Participants randomly allocated to the control arm will have access to the app and receive control instant messages about trial participation.

### Eligibility Criteria

Women aged 16 to 24 years who own a personal Android mobile phone and live in La Paz or El Alto, and who report an unmet need for contraception (ie, are sexually active, are not using effective contraception, and want to avoid a pregnancy), can provide informed consent, and can read Spanish will be eligible to take part. Participants must also be willing to receive messages about contraception on their mobile phone.

### Recruitment and Setting

To achieve a diverse sample, we will promote the trial through a variety of routes: CIES’s service delivery points in La Paz and El Alto, the CIES website, flyers distributed through CIES’s youth network, and social media sites. Potential participants will be provided the link to the enrollment pages of the secure online trial database and randomization system, where they can read the participant information sheet (Multimedia Appendix 1) and provide informed consent (Multimedia Appendix 2). (The information sheet and consent form will be provided to potential participants in Spanish. The English versions are included here for the purposes of publication.) If they do not have adequate Internet connectivity, youth network volunteers will provide this. Participants will also have the option of completing the paper-based version of the consent form.

To maximize the chance of recruiting to target, LSHTM conducted a pretrial training in La Paz to train local staff on all recruitment procedures. The training included discussions about the practicalities of recruitment with a view to developing the most appropriate strategies. CIES conducted a similar training with their youth volunteers, who will promote the trial.

**Figure 1:**
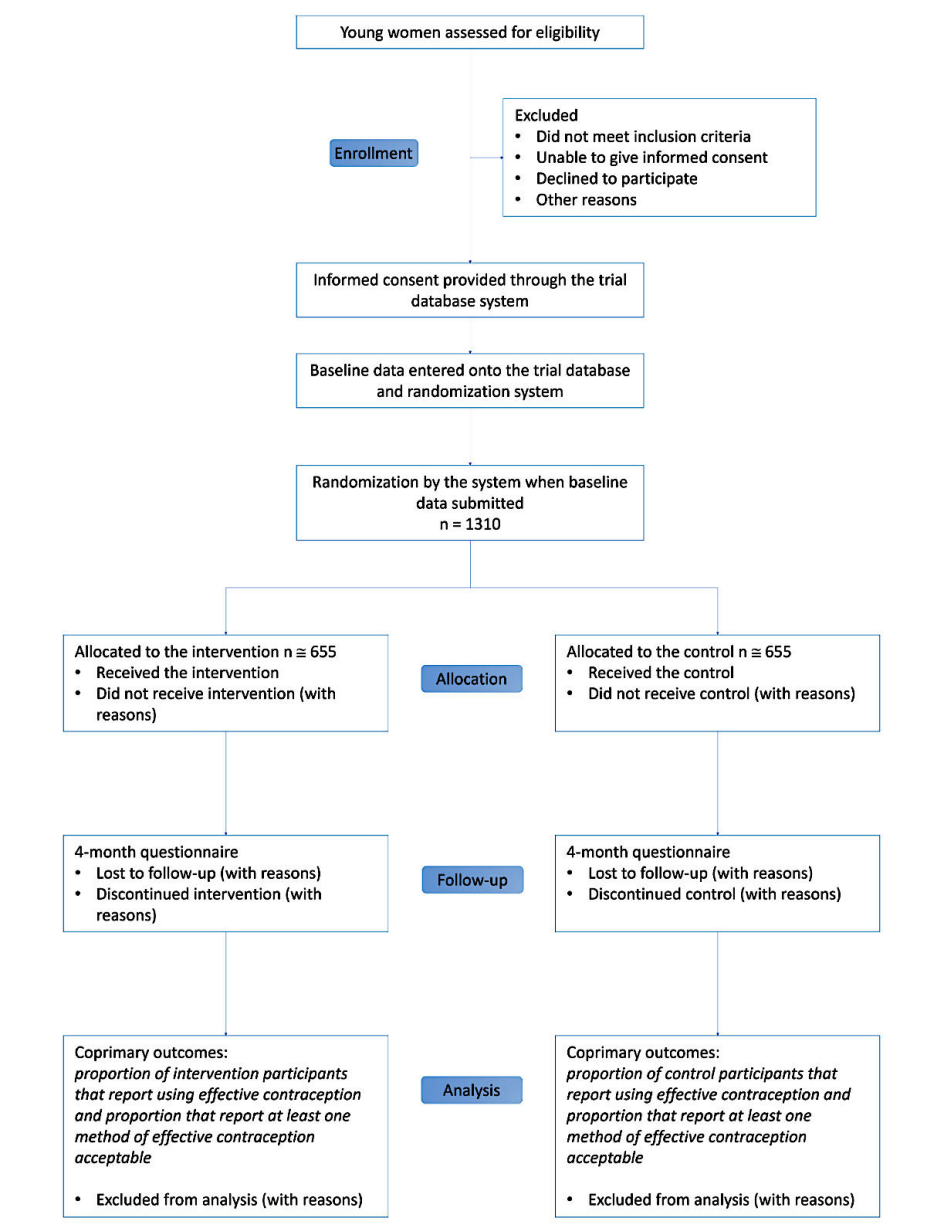
Consolidated Standards of Reporting Trials (CONSORT) flow diagram.

We will report the number of people assessed for eligibility, the number excluded before randomization, and the number of participants randomly allocated to the intervention, who completed follow-up, and who were analyzed (Figure 1).

### Intervention

In addition to providing accurate information about contraception (including the dual protection that condoms offer), the intervention messages target beliefs identified in the development phase that influence contraceptive use (eg, specific misconceptions about the side effects and health risks of contraception) and aim to support young women in believing that they can influence their reproductive health. The messages contain the following behavior change methods, adapted for delivery by mobile phone [50]: belief selection, facilitation, anticipated regret, guided practice, verbal persuasion, tailoring, cultural similarity, arguments, shifting perspective, and goal setting. The Tú decides app itself contains standard family planning information and no behavior change methods.

Participants allocated to the intervention group will receive 0 to 3 messages per day (a total of 183 messages) for 120 days. Included in the 183 messages that intervention recipients receive are 7 control messages about the importance of their participation and reminding them to contact the project coordinator if they change their number.

The message sets start with 6 days of messages with general information about the study, such as information about what they will receive over the next 120 days, how to stop the messages, who to contact if they change their number, how to keep the messages private, and information about who to call if they feel unsafe as a result of someone reading the messages.

On days 119 and 120, the intervention includes 4 messages: 1 that indicates that the messages have ended, 1 that provides a link to the database to complete the follow-up questionnaire, 1 that gives reassurance that the information they provide is confidential, and a final message stating that their participation is helping to determine the best ways to provide reproductive health services in Bolivia.

Details regarding the development and a description of the intervention will be reported in a forthcoming publication.

### Control

Participants allocated to the control group will have access to the same Tú decides app pages as the intervention group. Control participants will also receive 16 messages about trial participation over 120 days. The first 4 days include 6 messages that introduce the study, as well as providing information about what they will receive over the next 120 days, how to stop the messages, and who to contact if they change their number. They then receive 2 messages a month for 3 months: 1 about the importance of their participation and 1 reminding them to contact the project coordinator if they change their number. On day 105, they will receive 1 message about the importance of their participation. On day 120, participants will receive 3 messages: 1 that provides information on how to complete the follow-up questionnaire, 1 that gives reassurance that the information they provide is confidential, and a final message stating that their participation is helping to determine the best ways to provide reproductive health services in Bolivia.

All participants will receive usual care and will be free to seek any other support, whether existing or new.

### Outcomes

#### Primary Outcomes

The coprimary outcomes are self-reported current use of effective contraception and the proportion of participants reporting that at least one method of effective contraception is acceptable at 4 months after randomization. Because a validated measure of acceptability appropriate for this context did not exist, we constructed the primary outcome measure based on guidelines for measuring integrated behavioral model constructs [49,52,53] and tested its face validity with the target group. The acceptability of each method is measured by the following stems: “Using the [method]...causes infertility,...causes unwanted side effects,...is easy,...is a good way to prevent pregnancy” and “I would recommend the [method] to a friend.” Intrauterine device and implant acceptability is measured by an additional stem: “The [method] insertion would not be a problem for me.” The response options for each scale are “strongly disagree,” “disagree,” “not sure,” “agree,” “strongly agree,” and “I do not know what the [method] is.” A method is acceptable if participants report “agree” or “strongly agree” for all scales except for the “...causes infertility” and “...causes unwanted side effects” stems, for which “disagree” or “strongly disagree” denotes acceptability (items 1-27 in Multimedia Appendix 3 and items 4-30 in Multimedia Appendix 4).

#### Secondary Outcomes

Secondary outcomes are, for each contraception method, the proportion reporting that each effective contraception method is acceptable (acceptability of individual methods); the proportion reporting use of effective contraception at any time during the 4 months (discontinuation); the proportion reporting attending a sexual health service during the 4 months (service uptake); the proportion reporting that they became pregnant and they did not want to become pregnant during the study (unintended pregnancy); and the proportion reporting having an abortion during the study (induced abortion).

#### Process Outcomes

The process outcomes are knowledge of effective contraception; perceived norms and personal agency in relation to using contraception and communicating with partners about contraception; intention to use effective contraception; and intervention dose received.

### Data Collection

We will collect data at baseline and 4 months postrandomization using questionnaires. The questionnaires were written in English, translated into Spanish by a native speaker from Bolivia, and then tested for face validity with the target group. We asked 21 young women to comment on the length of the questionnaires, the comprehensibility of the questions, the meaning of the scales, and suggestions for improvement. All data will be entered onto the trial database system, which is on LSHTM’s secure server. At both time points, participants can fill out a paper-based version of the questionnaire at the recruitment site, provide the data over the phone with research staff, or enter data directly onto the online system, according to their preference. If participants provide their questionnaire data by paper or over the telephone, research staff will enter these data onto the system.

#### Baseline Data Collected

At baseline we will measure the acceptability of effective contraception (a coprimary outcome) and collect the following personal and demographic data via the baseline questionnaire: full name; mobile phone number; email address; date of birth; marital status; number of children; ethnicity; occupation; highest education level completed; residence; current method of contraception; and how they found out about the study (Multimedia Appendix 3).

#### Follow-Up Data Collected

At 4 months, we will measure the primary, secondary, and process outcomes and collect the following data via the follow-up questionnaire: if participants report using an effective method, where they obtained it; current pregnancy intention; whether they knew someone else who took part in the study and, if so, whether they read each other’s messages (contamination); whether they have experienced physical violence since being in the study; and whether anything good or bad happened as a result of receiving the messages (Multimedia Appendix 4). We are collecting data on physical violence because the intervention involves a sensitive topic and is delivered in a context where intimate partner violence is a public health concern. If participants do not complete the questionnaire themselves, local research staff will contact them to collect their data. For participants who report use of effective contraception on the follow-up questionnaire, local research staff will attempt to locate the service records to objectively verify use.

#### Methods to Maximize Follow-Up Response

**Figure 2:**
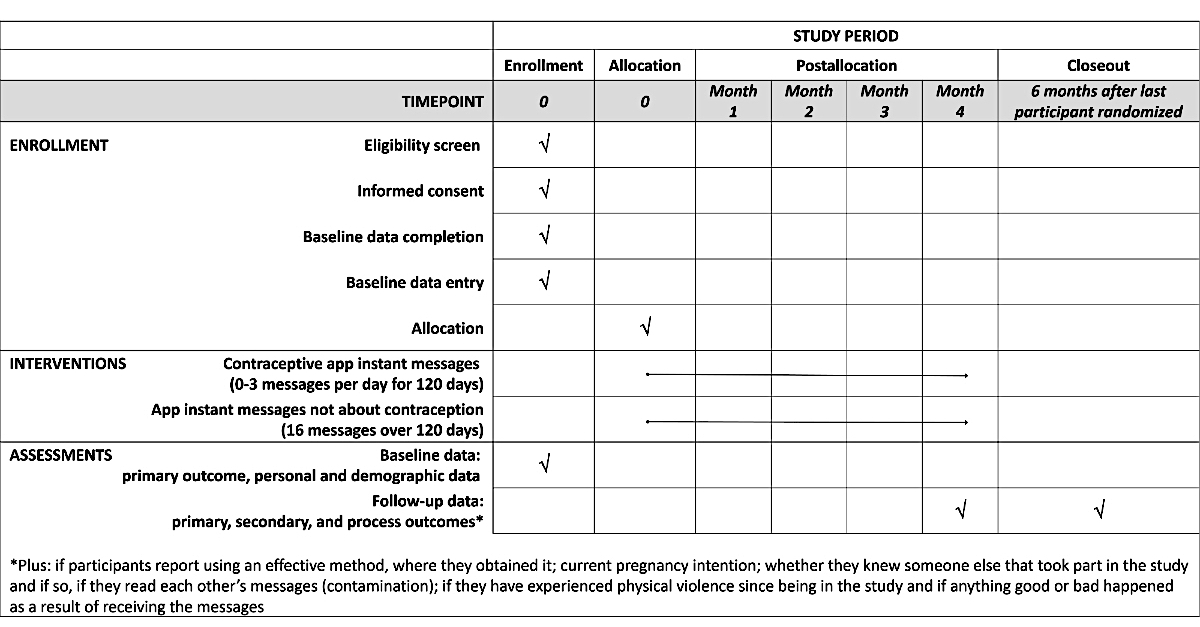
Schedule of enrollment, interventions, and assessments.

The pretrial training also included training in follow-up procedures. It emphasized the importance of ensuring that participants understand that participation involves completing a 4-month questionnaire and potentially receiving daily messages about contraception for 4 months. The control messages, also sent to participants allocated to the intervention, are an effort to keep participants engaged. Staff will contact nonresponders up to 3 times for their follow-up data. Follow-up will end 6 months after the last participant has been randomized or after staff has attempted to contact all nonresponders 3 times, whichever comes first. See Figure 2 for the schedule of enrollment, interventions, and assessments.

### Allocation and Protection Against Bias

Randomization will occur immediately after baseline data are submitted on the trial database and randomization system. The allocation sequence is generated by the remote computer-based randomization software, ensuring that investigators are unaware of allocation before participants are randomized. Due to the nature of the intervention, participants will be aware of the allocation soon after they start receiving the messages. Local research staff collecting outcome data will not be made aware of allocation unless this is revealed to them by the participant. Researchers who analyze the data will be masked to treatment allocation.

### Intervention Delivery

After participant baseline data have been entered, a confirmation of enrollment screen will provide instructions on how to install the app. When participants install the app, they will be prompted to enter the mobile phone number they entered on the baseline questionnaire. The trial database and randomization system will then send the allocation to the local app platform. Participants will then have access to the app and receive either the control or intervention messages, according to their allocation. Within the app, participants can choose when they want to receive the messages, and they can also stop the messages. Participants will receive the first message the day after they install the app.

### Sample Size

A trial evaluating a postabortion mobile phone intervention using voice messages and counsellor support found that 18% more women in the intervention arm than in the control arm were using effective contraception at 4 months (64% vs 46%, relative risk 1.39, 95% CI 1.17-1.66) [47]. Assuming that Smith and colleagues’ trial observed a larger increase in contraceptive uptake, as it involved women who had just had an abortion, we powered our trial to detect a smaller absolute difference of 10% uptake in effective contraception at 4 months.

The proportion of women aged 16 to 24 years in a partnership living in La Paz or El Alto using effective contraception is estimated to be around 44% [54]. A total of 1048 participants will allow us to have 90% power to detect a 10% increase in effective contraception, assuming 44% use in the control group (ie, 44% in the control vs 54% in the intervention, corresponding to an odds ratio of 1.49). Allowing for 20% loss to follow-up, we will randomly allocate 1310 people.

### Data Management

We did not convene a data monitoring and ethics committee, as the intervention provides support and is unlikely to produce adverse effects. We have convened a trial steering committee, and they have agreed to take on the monitoring of ethical aspects of the trial. The trial sponsor may audit the trial according to their own risk assessment and schedule.

Personal details entered onto the trial database and randomization system will be stored on LSHTM’s secure server. Personally identifiable information exported from the database will be stored separately from anonymized research data. Participant mobile phone numbers, but no other personal details, will be stored in the local platform that sends the messages through the app. Any signed paper consent forms and questionnaires will be kept in a data enclave at CIES. All data arising from the study will be kept confidential and accessible only to researchers directly involved in it. Personally identifiable data will not be kept longer than necessary and will be deleted within 3 months following study completion. We will retain primary research data for 10 years following study completion.

### Ethical Approval

The trial was granted ethical approval by LSHTM Interventions Research Ethics Committee on May 16, 2016 and by La Comisión de Ética de la Investigación del Comité Nacional de Bioética on September 20, 2016. The trial is registered by ClinicalTrials.gov (NCT02905526).

### Protocol Amendments

Any important changes to the protocol will be submitted to the LSHTM Interventions Research Ethics Committee as an amendment. Trial documentation will be updated accordingly and will be implemented once the Committee has approved the changes. LSHTM will communicate any changes relevant to local research staff.

### Dissemination

The research results will be cowritten by LSHTM and CIES and submitted for publication in peer-reviewed academic journals. We will adhere to the International Committee of Medical Journal Editors authorship criteria. We will disseminate findings to all the study stakeholders and policy makers in Bolivia.

### Analyses

#### General Statistical Considerations

The analysis of the data will follow the plan specified below. There will be no interim analyses and therefore no stopping rules. We will analyze participant data according to the arm that they were allocated to and will include only participants with complete outcome data in the primary analysis (a complete-case analysis). All statistical tests will be 2-sided. We will report all effect estimates with a 95% confidence interval and associated *P* value. Statistical significance will be considered at the 5% level, but interpreted with caution considering the 2 primary outcomes. We will use the latest version of Stata (StataCorp LLC) for analyses.

#### Loss to Follow-Up

To investigate whether loss to follow-up differs by arm, we will report this descriptively and use a chi-square test. We will use logistic regression to compare baseline characteristics of participants who completed 4-month follow-up against participants who did not. We will report predictors of loss to follow-up and investigate whether the effect of these differs by arm by testing for an interaction.

#### Assumptions About Missing Data

As we are not aware of similar trials, it is not possible to investigate the pattern of missing data. The complete-case analysis assumes that missing data for participants who did not complete follow-up are similar to data from participants who completed follow-up, conditionally on baseline covariates included in the analysis model (ie, that data are missing at random) [55]. If participants who complete follow-up are more likely to use effective contraception and to find an effective method acceptable compared with those who are lost to follow-up, the observed proportion may overestimate use and acceptability [55].

#### Missing Covariates

The database requires all items on the baseline questionnaire to be submitted in order to proceed to the random allocation. Therefore, there will be no missing baseline covariates.

#### Principal Analyses

##### Descriptive Analysis

We will report a flow diagram of trial participation, as recommended in the Consolidated Standards of Reporting Trials (CONSORT) guidelines [56]. We will report the baseline characteristics by treatment arm. We will also explore the baseline factors associated with retention (see above).

##### Analysis of the Primary Outcome

Both coprimary outcomes are binary, and we will compare the crude proportion who report using effective contraception in each group and the crude proportion who report that at least one method is acceptable in each group. We will estimate the difference between the groups using logistic regression and will report the odds ratio along with the 95% confidence interval and *P* value for evidence against the absence of intervention effect from the model. The primary analysis regression will be adjusted for baseline covariates likely to be associated with the outcome in order to improve the efficiency of the analysis and avoid chance imbalances [57]. The prespecified covariates that we will adjust for are age (16-19 years/20-24 years), number of children (0/≥1), highest education level completed (university/other), and acceptability of effective contraception at baseline (at least one method acceptable/no methods acceptable). Primary outcomes will be analyzed individually, and no formal multiplicity correction will be applied, but interpretation will take into account the multiple tests if only 1 of the 2 outcomes reaches the 5% significance level. We will also report the crude odds ratio between arms.

##### Analysis of the Secondary Outcomes

The analysis of the secondary outcomes will be the similar to the analysis of the primary outcome. We will estimate the difference between the groups using logistic regression, and report odds ratios with 95% confidence intervals and *P* values. All regressions will be adjusted for the prespecified covariates as above (although, with the acceptability of individual methods, the outcome at baseline will replace acceptability of effective contraception).

##### Analysis of the Process Outcomes

The process outcomes perceived norms, personal agency, and intention comprise ordinal scales. We will analyze each scale individually using ordered logistic regression to estimate proportional odds ratios. For knowledge, each correct answer will receive 1 point. The points will be summed and an overall score will be produced. We will use linear regression to test for a difference in mean scores between the arms.

To assess the “dose” of the intervention that the intervention participants received, we will analyze the number of messages that participants reported to have read (all, most, some, none) and whether they stopped the messages. We will report this descriptively.

#### Additional Analyses

##### Sensitivity Analyses

We will conduct sensitivity analyses regarding the missing data. In the first sensitivity analysis, we will consider that data are not missing at random; that participants lost to follow-up did not find at least one method acceptable; and that participants lost to follow-up were not using an effective method of contraception. In the second, we will adjust for the main baseline predictors of missingness. Both sensitivity analyses will be adjusted for the prespecified covariates as above.

##### Subgroup Analysis

Recognizing that the trial is not powered to detect effect differences in subgroups, we will conduct exploratory subgroup analyses for the coprimary outcomes to determine whether the intervention effect varies by baseline characteristics. The prespecified subgroups are age (split at the median); marital status (married/not married); number of children (0/≥1); geographic location (El Alto/La Paz); occupation (in education/other); and highest education level completed (university/other). Within the prespecified subgroups, we will assess heterogeneity of treatment effect with a test for interaction [58-62]. Interaction test *P* values will be presented but will be interpreted with caution, due to the exploratory nature, the multiple tests performed, and the low power of the interaction test. We will estimate odds ratios along with 95% CIs for each subgroup without *P* values. As this is an exploratory analysis of potentially influential characteristics that are not justified a priori, we will not hypothesize effect directions.

##### Contamination

To assess the potential for contamination, we will report the proportion of control group participants who read another participant’s messages and the proportion of intervention participants whose messages were read by another participant.

##### Analysis of Pooled Trial Data

We are conducting trials of a similar intervention in 2 other countries. If the results of the other trials are available, we will conduct the principal analyses on the pooled dataset.

The datasets used and analyzed during this study are available from the corresponding author on reasonable request.

### Participants’ Rights and Safety

Participants will have the right to withdraw at any time during their involvement, without having to give a reason. Participants can withdraw by contacting the project coordinator. Acting on a participant’s request to withdraw from the trial, we will change the participant’s status to “withdrawn” and exclude the person from the list of participants who are due for follow-up. Participants’ participation and personal identifiable data will remain confidential and research data will be anonymized.

In the formative work, we explored young people’s views on confidentiality about receiving messages on their mobile phone. While the majority of participants did not report concerns regarding receiving messages about contraception on their mobile phone, it is possible that some participants will want to keep the messages confidential from certain people (eg, partner, parents) and that these people might view the messages. The messages remind participants that they can delete the messages and provide instructions on how to keep the messages private. We will provide participants with information on support services that they can contact if they feel unsafe as a consequence of the messages being read. We will review physical violence during participants’ involvement in the trial reported on the follow-up questionnaire.

## Results

Recruitment commenced on March 1, 2017 and was completed on July 29, 2017. The estimated completion date for the final participant recruited (final data collection date for the primary outcome) is January 2018.

## Discussion

Among young women in La Paz and El Alto with an unmet need, the results of this trial will provide evidence for the effect of the intervention on their use of and attitudes toward effective contraception. The analysis of the secondary and process outcomes may provide evidence for the effect of the intervention on attitudes toward the individual effective methods, service use, unintended pregnancy, induced abortion, and the psychological processes that may have been altered by the intervention.

Because this trial is being conducted among young women with an unmet need for contraception in a context where information about contraception is low, it is reasonable to assume that enrolling in the trial will be popular. While this is an advantage with regard to meeting the recruitment target, it is possible that participants will tell their friends about the trial and that they will also enroll. While this is desired in a nontrial context, this could lead to contamination if the intervention messages are shared with control participants during the trial. To minimize this, participants will not be recruited through schools.

Because the intervention is being delivered through the Tú decides app, participants must own a personal Android mobile phone to take part in the trial. Although the intervention development work indicated that the majority of young people in Bolivia own a personal Android mobile phone, not everyone in the target group will. It may be that young people less likely to use and to find contraceptive methods acceptable are more likely to not own an Android phone, which would limit the generalizability of the findings. Smartphone ownership continues to increase rapidly, however, so it is likely that a greater proportion of young people from different socioeconomic communities will be able to receive the intervention in the future.

The trial will assess the effect of sending instant messages containing behavior change methods in addition to the app; it is not assessing the effect of the app itself. It is possible that the app, which provides standard family planning information, could have an effect on effective contraceptive use and acceptability of effective contraception. If the app itself is very effective, the added benefit of the instant messages will be lower. The results of the study will add to current research on mobile phones for intervention delivery and will determine whether mobile phones can be an important adjunct to sexual and reproductive health service provision in Bolivia.

## Appendix

Multimedia Appendix 1 Trial information sheet.

Multimedia Appendix 2 Trial consent form.

Multimedia Appendix 3 Baseline questionnaire.

Multimedia Appendix 4 Follow-up questionnaire.

## Abbreviations

CIES: Centro de Investigación, Educación y Servicios
CONSORT: Consolidated Standards of Reporting Trials
IPPF: International Planned Parenthood Federation
LSHTM: London School of Hygiene & Tropical Medicine
