# Supplementary material for: An Intervention Delivered by App Instant Messaging to Increase Acceptability and Use of Effective Contraception Among Young Women in Bolivia: Protocol of a Randomized Controlled Trial
Source: JMIR Res Protoc. 2017 Dec 18;6(12):e252. doi: 10.2196/resprot.8679 (PMC5748473; doi:10.2196/resprot.8679)
Supplement: Multimedia Appendix 2 [file resprot_v6i12e252_app2.pdf]

**Project Coordinator**: Maria Eugenia Torrico, CIES Salud Sexual - Salud Reproductiva, Calle 6 de Obrajes Nro. 614 – Casilla 9935, (591-2) 2788162, maeugenia@cies.org.bo

**Principal Investigator**: Ona McCarthy, The London School of Hygiene and Tropical Medicine, Department of Population Health, Keppel St, WC1E 7HT, London, United Kingdom, ona.mccarthy@lshtm.ac.uk

**Please initial here**

1. I have read the Information sheet for the above study (v3 02.11.16) or it has been read to me. I have had the opportunity to consider the information in it. ☐

2. I have had the opportunity to ask questions and I am happy with the answers that you gave me. ☐

3. I understand that I do not have to take part if I do not want to. ☐

4. I understand that the information that I provide will remain confidential and will only be used for this study. Only the research team will have access to this information. ☐

5. I agree to the study researchers searching CIES clinic records to check for any services that I may have received during my participation in the study. ☐

6. I understand that I am free to leave the study at any time without having to give a reason. I understand that this will not affect the services that I receive. ☐

7. **I consent to take part in the above study.** ☐

*(optional) I agree to be contacted about taking part in a short interview* ☐

| ____________________ | ____________________ | ________ |
|---|---|---|
| Name of participant (print) | Signature of participant | Date |

**Statement by person taking consent**

I confirm that the participant was given an opportunity to ask questions about the study, and all the questions asked by the participant have been answered correctly and to the best of my ability. I confirm that the individual has not been coerced into giving consent, and the consent has been given freely and voluntarily. A copy of the consent form has been given to the participant.

| ____________________ | ____________________ | ________ |
|---|---|---|
| Name of person taking consent (print) | Signature of person taking consent | Date |
