# Supplementary material for: An Intervention Delivered by App Instant Messaging to Increase Acceptability and Use of Effective Contraception Among Young Women in Bolivia: Protocol of a Randomized Controlled Trial
Source: JMIR Res Protoc. 2017 Dec 18;6(12):e252. doi: 10.2196/resprot.8679 (PMC5748473; doi:10.2196/resprot.8679)
Supplement: Multimedia Appendix 3 [file resprot_v6i12e252_app3.pdf]

Thank you very much for taking part in the study. Please complete the following questionnaire. Please be as honest as possible. All of your answers will remain confidential.							
	Using the pill…						
1	…causes infertility	Strongly disagree	Disagree	Not sure	Agree	Strongly agree	I do not know what the pill is
2	…causes unwanted side-effects	Strongly disagree	Disagree	Not sure	Agree	Strongly agree	I do not know what the pill is
3	…is easy	Strongly disagree	Disagree	Not sure	Agree	Strongly agree	I do not know what the pill is
4	…is a good way to prevent pregnancy	Strongly disagree	Disagree	Not sure	Agree	Strongly agree	I do not know what the pill is
5	I would recommend the pill to a friend	Strongly disagree	Disagree	Not sure	Agree	Strongly agree	I do not know what the pill is
	Using the IUD…						
6	…causes infertility	Strongly disagree	Disagree	Not sure	Agree	Strongly agree	I do not know what the IUD is
7	…causes unwanted side-effects	Strongly disagree	Disagree	Not sure	Agree	Strongly agree	I do not know what the IUD is
8	…is easy	Strongly disagree	Disagree	Not sure	Agree	Strongly agree	I do not know what the IUD is
9	…is a good way to prevent pregnancy	Strongly disagree	Disagree	Not sure	Agree	Strongly agree	I do not know what the IUD is
10	I would recommend the IUD to a friend	Strongly disagree	Disagree	Not sure	Agree	Strongly agree	I do not know what the IUD is
11	The IUD insertion would not be a problem	Strongly disagree	Disagree	Not sure	Agree	Strongly agree	I do not know what the IUD is
	Using the injection…						
12	…causes infertility	Strongly disagree	Disagree	Not sure	Agree	Strongly agree	I do not know what the injection is
13	…causes unwanted side-effects	Strongly disagree	Disagree	Not sure	Agree	Strongly agree	I do not know what the injection is
14	…is easy	Strongly disagree	Disagree	Not sure	Agree	Strongly agree	I do not know what the injection is
15	…is a good way to prevent pregnancy	Strongly disagree	Disagree	Not sure	Agree	Strongly agree	I do not know what the injection is
16	I would recommend the injection to a friend	Strongly disagree	Disagree	Not sure	Agree	Strongly agree	I do not know what the injection is
	Using the implant…						
17	…causes infertility	Strongly disagree	Disagree	Not sure	Agree	Strongly agree	I do not know what the implant is
18	…causes unwanted side-effects	Strongly disagree	Disagree	Not sure	Agree	Strongly agree	I do not know what the implant is
19	…is easy	Strongly disagree	Disagree	Not sure	Agree	Strongly agree	I do not know what the implant is
20	…is a good way to prevent pregnancy	Strongly disagree	Disagree	Not sure	Agree	Strongly agree	I do not know what the implant is
21	I would recommend the implant to a friend	Strongly disagree	Disagree	Not sure	Agree	Strongly agree	I do not know what the implant is
22	The implant insertion would not be a problem	Strongly disagree	Disagree	Not sure	Agree	Strongly agree	I do not know what the implant is

	Using the patch…						
23	…causes infertility	Strongly disagree	Disagree	Not sure	Agree	Strongly agree	I do not know what the patch is
24	…causes unwanted side-effects	Strongly disagree	Disagree	Not sure	Agree	Strongly agree	I do not know what the patch is
25	…is easy	Strongly disagree	Disagree	Not sure	Agree	Strongly agree	I do not know what the patch is
26	…is a good way to prevent pregnancy	Strongly disagree	Disagree	Not sure	Agree	Strongly agree	I do not know what the patch is
27	I would recommend the patch to a friend	Strongly disagree	Disagree	Not sure	Agree	Strongly agree	I do not know what the patch is

28	What is your first name?						
29	What is your last name?						
30	What is your mobile number?						
31	What is your email address?						
32	What day were you born?	Day	Month	Year			
33	Are you?	Married	Not married				
34	How many children do you have?	0	1	2+			
35	You identify with some indigenous origin, mark as appropriate	Aymara	Quechua	Guarani	Ninguno	Otro	
36	Are you? (check all that apply)	At school	At university	Working	Training	Not working	
37	What is the highest level of education that you have completed?	Primary	Secondary	University	Technical education		
38	Where do you live?	El Alto	Norte La Paz	Central La Paz	Sur La Paz		
39	What method of contraception are you or your partner using now (check all that apply)?	None		Male condom			
				Female condom	Calendar-based method	LAM	
		Withdrawal			Other method		
40	How did you find out about this study?	Facebook	OK	CIES	TFPA website	Friend	Flyer/poster
							Otro

Muchas gracias por participar en el estudio. Por favor complete el siguiente cuestionario. Por favor sea lo más honesta posible. Todas sus respuestas serán confidenciales.

	Usar las pastillas….						

1	...causa infertifilidad.	Muy en desacuerdo	Desacuerdo	No lo sé	De acuerdo	Muy de acuerdo	No sé lo que son las pastillas
2	…tiene efectos secundarios no agradables.	Muy en desacuerdo	Desacuerdo	No lo sé	De acuerdo	Muy de acuerdo	No sé lo que son las pastillas
3	… es fácil de usar.	Muy en desacuerdo	Desacuerdo	No lo sé	De acuerdo	Muy de acuerdo	No sé lo que son las pastillas
4	… es un buen método para prevenir embarazos.	Muy en desacuerdo	Desacuerdo	No lo sé	De acuerdo	Muy de acuerdo	No sé lo que son las pastillas
5	Yo recomendaría el uso de pastillas a una amiga.	Muy en desacuerdo	Desacuerdo	No lo sé	De acuerdo	Muy de acuerdo	No sé lo que son las pastillas
	Usar la t de cobre…						
6	...causa infertifilidad.	Muy en desacuerdo	Desacuerdo	No lo sé	De acuerdo	Muy de acuerdo	No sé lo que es la t de cobre
7	…tiene efectos secundarios no agradables.	Muy en desacuerdo	Desacuerdo	No lo sé	De acuerdo	Muy de acuerdo	No sé lo que es la t de cobre
8	…es fácil de usar.	Muy en desacuerdo	Desacuerdo	No lo sé	De acuerdo	Muy de acuerdo	No sé lo que es la t de cobre
9	… es un buen método para prevenir embarazos.	Muy en desacuerdo	Desacuerdo	No lo sé	De acuerdo	Muy de acuerdo	No sé lo que es la t de cobre
10	Yo recomendaría el uso de la t de cobre a una amiga.	Muy en desacuerdo	Desacuerdo	No lo sé	De acuerdo	Muy de acuerdo	No sé lo que es la t de cobre
11	La inserción de la T o la forma de colocarla no representa un problema.	Muy en desacuerdo	Desacuerdo	No lo sé	De acuerdo	Muy de acuerdo	No sé lo que es la t de cobre
	Usar la inyección …						
12	...causa infertifilidad.	Muy en desacuerdo	Desacuerdo	No lo sé	De acuerdo	Muy de acuerdo	No sé lo que son las inyecciones
13	…tiene efectos secundarios no agradables.	Muy en desacuerdo	Desacuerdo	No lo sé	De acuerdo	Muy de acuerdo	No sé lo que son las inyecciones
14	…es fácil de usar.	Muy en desacuerdo	Desacuerdo	No lo sé	De acuerdo	Muy de acuerdo	No sé lo que son las inyecciones
15	… es un buen método para prevenir embarazos.	Muy en desacuerdo	Desacuerdo	No lo sé	De acuerdo	Muy de acuerdo	No sé lo que son las inyecciones
16	Yo recomendaría el uso de la inyección a una amiga.	Muy en desacuerdo	Desacuerdo	No lo sé	De acuerdo	Muy de acuerdo	No sé lo que son las inyecciones
	Usar el implante…						
17	...causa infertifilidad.	Muy en desacuerdo	Desacuerdo	No lo sé	De acuerdo	Muy de acuerdo	No sé lo que es el implante
18	…tiene efectos secundarios no agradables.	Muy en desacuerdo	Desacuerdo	No lo sé	De acuerdo	Muy de acuerdo	No sé lo que es el implante
19	…es fácil de usar.	Muy en desacuerdo	Desacuerdo	No lo sé	De acuerdo	Muy de acuerdo	No sé lo que es el implante
20	… es un buen método para prevenir embarazos.	Muy en desacuerdo	Desacuerdo	No lo sé	De acuerdo	Muy de acuerdo	No sé lo que es el implante
21	Yo recomendaría el uso del implante a una amiga.	Muy en desacuerdo	Desacuerdo	No lo sé	De acuerdo	Muy de acuerdo	No sé lo que es el implante

22	La inserción del implante o la forma de colocarlo no representa un problema.	Muy en desacuerdo	Desacuerdo	No lo sé	De acuerdo	Muy de acuerdo	No sé lo que es el implante
	Uso del parche…						
23	...causa infertifilidad.	Muy en desacuerdo	Desacuerdo	No lo sé	De acuerdo	Muy de acuerdo	No sé lo que es el parche
24	…tiene efectos secundarios no agradables.	Muy en desacuerdo	Desacuerdo	No lo sé	De acuerdo	Muy de acuerdo	No sé lo que es el parche
25	…es fácil de usar.	Muy en desacuerdo	Desacuerdo	No lo sé	De acuerdo	Muy de acuerdo	No sé lo que es el parche
26	… es un buen método para prevenir embarazos.	Muy en desacuerdo	Desacuerdo	No lo sé	De acuerdo	Muy de acuerdo	No sé lo que es el parche
27	Yo recomendaría el uso del parche a una amiga.	Muy en desacuerdo	Desacuerdo	No lo sé	De acuerdo	Muy de acuerdo	No sé lo que es el parche

28	¿Cuál es tu nombre?						
29	¿Cuál es tu apellido?						
30	¿Cuál es tu número de celular?						
31	¿Cuál es tu dirección de correo electrónico?						
32	¿Qué fecha naciste?	Día	Mes	Año			
33	¿Cuál es tu estado civil?	Casada	No casada				
34	¿Cuántos hijos tienes?	0	1	2+			
35	Te identificas con algún Pueblo Indígena originario	Aymara	Quechua	Guarani	Ninguno	Otro	
36	Tú estás (marque todo lo que corresponda)	En la escuela	En la Universidad	Trabajando	Capacitándose	Sin trabajo	
37	¿Cuál es el máximo nivel educativo que has completado?	Primario	Secundario	Universitario	Técnico		
38	¿Dónde vives?	El Alto	La Paz zona norte	La Paz zone central	La Paz zona sur		
39	¿Qué método anticonceptivo está utilizando <u>actualmente</u> (marque todos lo que correspondan)?	Ninguno	Condón masculino	Método de lactancia			
		Condón femenino	Calendario	Otro método			
		Retiro					
40	¿Cómo ha conocido este estudio?	Facebook	CIES El Alto	CIES La Paz	CIES sitio web	Amigo	Volantes/póster
							Otro
