# Supplementary material for: An Intervention Delivered by App Instant Messaging to Increase Acceptability and Use of Effective Contraception Among Young Women in Bolivia: Protocol of a Randomized Controlled Trial
Source: JMIR Res Protoc. 2017 Dec 18;6(12):e252. doi: 10.2196/resprot.8679 (PMC5748473; doi:10.2196/resprot.8679)
Supplement: Multimedia Appendix 4 [file resprot_v6i12e252_app4.pdf]

Thank you very much for taking part in the study. Now it is time to complete the final questionnaire. Please be as honest as possible. All of your answers will remain confidential.						
1	What method of contraception are you using now (check all that apply)?	None	Injection	Male condom	IUD	Not sexually active
		Implant	Pill	Female condom	Calendar-based method	LAM
		Withdrawal	Patch	Ring	Other method	
2	Where did you get this method?	CIES El Alto	CIES La Paz	Other service	Not using a method	I do not know
3	Do you want a pregnancy now?	Yes	No	Not sure	Not sexually active	

	Using the pill…						
4	…causes infertility	Strongly disagree	Disagree	Not sure	Agree	Strongly agree	I do not know what the pill is
5	…causes unwanted side-effects	Strongly disagree	Disagree	Not sure	Agree	Strongly agree	I do not know what the pill is
6	…is easy	Strongly disagree	Disagree	Not sure	Agree	Strongly agree	I do not know what the pill is
7	…is a good way to prevent pregnancy	Strongly disagree	Disagree	Not sure	Agree	Strongly agree	I do not know what the pill is
8	I would recommend the pill to a friend	Strongly disagree	Disagree	Not sure	Agree	Strongly agree	I do not know what the pill is
	Using the IUD…						
9	…causes infertility	Strongly disagree	Disagree	Not sure	Agree	Strongly agree	I do not know what the IUD is
10	…causes unwanted side-effects	Strongly disagree	Disagree	Not sure	Agree	Strongly agree	I do not know what the IUD is
11	…is easy	Strongly disagree	Disagree	Not sure	Agree	Strongly agree	I do not know what the IUD is
12	…is a good way to prevent pregnancy	Strongly disagree	Disagree	Not sure	Agree	Strongly agree	I do not know what the IUD is
13	I would recommend the IUD to a friend	Strongly disagree	Disagree	Not sure	Agree	Strongly agree	I do not know what the IUD is
14	The IUD insertion would not be a problem	Strongly disagree	Disagree	Not sure	Agree	Strongly agree	I do not know what the IUD is
	Using the injection…						
15	…causes infertility	Strongly disagree	Disagree	Not sure	Agree	Strongly agree	I do not know what the injection is
16	…causes unwanted side-effects	Strongly disagree	Disagree	Not sure	Agree	Strongly agree	I do not know what the injection is
17	…is easy	Strongly disagree	Disagree	Not sure	Agree	Strongly agree	I do not know what the injection is
18	…is a good way to prevent pregnancy	Strongly disagree	Disagree	Not sure	Agree	Strongly agree	I do not know what the injection is
19	I would recommend the injection to a friend	Strongly disagree	Disagree	Not sure	Agree	Strongly agree	I do not know what the injection is
	Using the implant…						

20	…causes infertility	Strongly disagree	Disagree	Not sure	Agree	Strongly agree	I do not know what the implant is
21	…causes unwanted side-effects	Strongly disagree	Disagree	Not sure	Agree	Strongly agree	I do not know what the implant is
22	…is easy	Strongly disagree	Disagree	Not sure	Agree	Strongly agree	I do not know what the implant is
23	…is a good way to prevent pregnancy	Strongly disagree	Disagree	Not sure	Agree	Strongly agree	I do not know what the implant is
24	I would recommend the implant to a friend	Strongly disagree	Disagree	Not sure	Agree	Strongly agree	I do not know what the implant is
25	The implant insertion would not be a problem	Strongly disagree	Disagree	Not sure	Agree	Strongly agree	I do not know what the implant is
	Using the patch…						
26	…causes infertility	Strongly disagree	Disagree	Not sure	Agree	Strongly agree	I do not know what the patch is
27	…causes unwanted side-effects	Strongly disagree	Disagree	Not sure	Agree	Strongly agree	I do not know what the patch is
28	…is easy	Strongly disagree	Disagree	Not sure	Agree	Strongly agree	I do not know what the patch is
29	…is a good way to prevent pregnancy	Strongly disagree	Disagree	Not sure	Agree	Strongly agree	I do not know what the patch is
30	I would recommend the patch to a friend	Strongly disagree	Disagree	Not sure	Agree	Strongly agree	I do not know what the patch is

31	Have you become pregnant since joining this study?	Yes	No	Not sexually active		
32	Did you want to become during this study?	Yes	No	Not sure	Not sexually active	
33	Have you had a termination (abortion) since joining this study?	Yes	No	Not sexually active		
34	What method of contraception have you used during this study (check all that apply)?	None	Injection	Male condom	IUD	Not sexually active
		Implant	Pill	Female condom	Calendar-based method	LAM
		Withdrawal	Patch	Ring	Other method	
35	How many times have you attended a sexual health service since you joined the study 4 months ago?	0	1	2+		
36	How many messages did you read?	All	Most	Some	None	
37	Did you stop the messages?	Yes	No			
38	Do you know anyone else in the study?	Yes	No			
39	Did they read the messages that we sent you?	Yes	No	I do not know anyone in the study		
40	Did you read the messages that we sent them?	Yes	No	I do not know anyone in the study		
41		Yes	No			

	Have you experienced physical violence since being in the study?					
42	Did anything good or bad happen as a result of receiving the messages? If so, please summarise here:					

43	Hormonal contraception is more effective at preventing pregnancy than condoms alone	Disagree	Agree	Do not know		
44	The pill is taken once a month	Disagree	Agree	Do not know		
45	The IUD lasts for 6 months	Disagree	Agree	Do not know		
46	The injection is given once a year	Disagree	Agree	Do not know		
47	The implant can stay under the skin for 10 years	Disagree	Agree	Do not know		
48	A new patch is worn each week for 3 weeks, then no patch for the 4th week	Disagree	Agree	Do not know		
49	I know where to get contraception	Disagree	Agree	Do not know		

50	My friends would use the pill, IUD, injection, implant or patch if they wanted to prevent pregnancy.	Strongly disagree	Disagree	Not sure	Agree	Strongly agree
51	My friends would talk to their partner about contraception if they wanted to prevent a pregnancy.	Strongly disagree	Disagree	Not sure	Agree	Strongly agree

52	If you wanted to use the pill, IUD, injection, implant or patch, how easy would it be for you to use it?	Very difficult	Difficult	Not sure	Easy	Very easy
53	If you wanted to talk to your partner about contraception, how easy would it be for you to talk to him?	Very difficult	Difficult	Not sure	Easy	Very easy

54	If you wanted to use the pill, IUD, injection, implant or patch, how certain are you that you could use it?	Very certain I could not	Certain I could not	Not sure	Certain I could	Very certain I could
55	If you wanted to talk to your partner about contraception, how certain are you that you could talk to him?	Very certain I could not	Certain I could not	Not sure	Certain I could	Very certain I could

56	I intend to use the pill, IUD, injection, implant or patch	Strongly disagree	Disagree	Not sure	Agree	Strongly agree

**Muchas gracias por haber participado en el estudio. Ahora es el momento de completar el cuestionario final. Por favor sea lo más honesta posible. Todas sus respuestas serán confidenciales**						
1	¿Qué método anticonceptivo estás utilizando actualmente (marca todos lo que correspondan)?	Ninguno	Inyección	Condón masculino	t de cobre	No eres sexualmente activa
		Implante	Píldora	Condón femenino	Calendario	Método de lactancia
		Retiro	Parche anticonceptivo	Anillo Vaginal	Otro método	

2	¿Dónde obtuviste el método?	CIES El Alto	CIES La Paz	Otro servicio	No está utilizando un método	No lo sé
3	¿Quieres embarazarte en este momento?	Sí	No	No estas segura	No eres sexualmente activa	

	Usar las pastillas….						
4	...causa infertifilidad.	Muy en desacuerdo	Desacuerdo	No lo sé	De acuerdo	Muy de acuerdo	No sé lo que son las pastillas
5	…tiene efectos secundarios desagradables.	Muy en desacuerdo	Desacuerdo	No lo sé	De acuerdo	Muy de acuerdo	No sé lo que son las pastillas
6	… es fácil de usar.	Muy en desacuerdo	Desacuerdo	No lo sé	De acuerdo	Muy de acuerdo	No sé lo que son las pastillas
7	…es una buena forma de prevenir embarazos.	Muy en desacuerdo	Desacuerdo	No lo sé	De acuerdo	Muy de acuerdo	No sé lo que son las pastillas
8	Yo le recomendaría el uso de pastillas a una amiga.	Muy en desacuerdo	Desacuerdo	No lo sé	De acuerdo	Muy de acuerdo	No sé lo que son las pastillas
	Usar la t de cobre …						
9	...causa infertifilidad.	Muy en desacuerdo	Desacuerdo	No lo sé	De acuerdo	Muy de acuerdo	No sé lo que es la t de cobre.
10	…tiene efectos secundarios desagradables.	Muy en desacuerdo	Desacuerdo	No lo sé	De acuerdo	Muy de acuerdo	No sé lo que es la t de cobre.
11	… es fácil de usar.	Muy en desacuerdo	Desacuerdo	No lo sé	De acuerdo	Muy de acuerdo	No sé lo que es la t de cobre.
12	…es una buena forma de prevenir embarazos.	Muy en desacuerdo	Desacuerdo	No lo sé	De acuerdo	Muy de acuerdo	No sé lo que es la t de cobre.
13	Yo le recomendaría el uso de la t de cobre a una amiga.	Muy en desacuerdo	Desacuerdo	No lo sé	De acuerdo	Muy de acuerdo	No sé lo que es la t de cobre.
14	La inserción de la t de cobre no es un problema	Muy en desacuerdo	Desacuerdo	No lo sé	De acuerdo	Muy de acuerdo	No sé lo que es la t de cobre.
	Usar la inyección …						
15	...causa infertifilidad.	Muy en desacuerdo	Desacuerdo	No lo sé	De acuerdo	Muy de acuerdo	No sé lo que son las inyecciones.
16	…tiene efectos secundarios desagradables.	Muy en desacuerdo	Desacuerdo	No lo sé	De acuerdo	Muy de acuerdo	No sé lo que son las inyecciones.
17	… es fácil de usar.	Muy en desacuerdo	Desacuerdo	No lo sé	De acuerdo	Muy de acuerdo	No sé lo que son las inyecciones.

18	…es una buena forma de prevenir embarazos.	Muy en desacuerdo	Desacuerdo	No lo sé	De acuerdo	Muy de acuerdo	No sé lo que son las inyecciones.
19	Yo le recomendaría el uso de la inyección a una amiga.	Muy en desacuerdo	Desacuerdo	No lo sé	De acuerdo	Muy de acuerdo	No sé lo que son las inyecciones.
	Usar el implante …						
20	...causa infertifilidad.	Muy en desacuerdo	Desacuerdo	No lo sé	De acuerdo	Muy de acuerdo	No sé lo que es el implante.
21	…tiene efectos secundarios desagradables.	Muy en desacuerdo	Desacuerdo	No lo sé	De acuerdo	Muy de acuerdo	No sé lo que es el implante.
22	… es fácil de usar.	Muy en desacuerdo	Desacuerdo	No lo sé	De acuerdo	Muy de acuerdo	No sé lo que es el implante.
23	…es una buena forma de prevenir embarazos.	Muy en desacuerdo	Desacuerdo	No lo sé	De acuerdo	Muy de acuerdo	No sé lo que es el implante.
24	Yo le recomendaría el uso del implante a una amiga.	Muy en desacuerdo	Desacuerdo	No lo sé	De acuerdo	Muy de acuerdo	No sé lo que es el implante.
25	La inserción del implante no es un problema	Muy en desacuerdo	Desacuerdo	No lo sé	De acuerdo	Muy de acuerdo	No sé lo que es el implante.
	Uso del parche …						
26	...causa infertifilidad.	Muy en desacuerdo	Desacuerdo	No lo sé	De acuerdo	Muy de acuerdo	No sé lo que es el parche.
27	…tiene efectos secundarios desagradables.	Muy en desacuerdo	Desacuerdo	No lo sé	De acuerdo	Muy de acuerdo	No sé lo que es el parche.
28	… es fácil de usar.	Muy en desacuerdo	Desacuerdo	No lo sé	De acuerdo	Muy de acuerdo	No sé lo que es el parche.
29	…es una buena forma de prevenir embarazos.	Muy en desacuerdo	Desacuerdo	No lo sé	De acuerdo	Muy de acuerdo	No sé lo que es el parche.
30	Yo le recomendaría el uso del parche a una amiga.	Muy en desacuerdo	Desacuerdo	No lo sé	De acuerdo	Muy de acuerdo	No sé lo que es el parche.

31	¿Durante el tiempo que duro el estudio quedaste embarazada?	Sí	No			
32	¿Planeabas quedar embarazada durante este estudio?	Sí	No	No estoy sergura	No eres sexualmente activa	
33	¿Has tenido una interrupción del embarazo (aborto) desde que te uniste al estudio?	Sí	No	No eres sexualmente activa		
34	¿Qué método anticonceptivo hasutilizado durante el estudio	Ninguno	Inyección	Condón masculino	t de cobre	No eressexualmente activa

	(marca todos los que correspondan)?	Implante	Píldora	Condón femenino	Calendario	Método de lactancia
		Retiro	Parche anticonceptivo	Anillo Vaginal	Otro método	
35	¿Cuántas veces has asistido a un servicio de salud sexual desde que te uniste al estudio, hace 4 meses?	0	1	2+		
36	¿Cuántos mensajes has leído?	Todos	La mayoría	Algunos	Ninguno	
37	¿Interrumpiste la llegada de los mensajes?	Sí	No			
38	¿Conoces a alguien más que haya participado en el estudio?	Sí	No			
39	¿Estas personas leyeron los mensajes que te enviamos?	Sí	No	No conozco a nadie en el estudio		
40	¿Leiste los mensajes que les enviamos a ellas?	Sí	No	No conozco a nadie en el estudio		
41	¿Has sufrido violencia física durante el period del etudio?	Sí	No			
42	¿Sucedió algo bueno o malo a consecuencia de recibir los mensajes? Si es así, por favor escríbelo aquí.					

43	La anticoncepción hormonal es más efectiva previniendo embarazos que los condones.	Desacuerdo	De acuerdo	No lo sé
44	Las pastillas anticonceptivas se toman una vez al mes.	Desacuerdo	De acuerdo	No lo sé
45	La t de cobre dura 6 meses	Desacuerdo	De acuerdo	No lo sé
46	La inyección se pone una vez al año.	Desacuerdo	De acuerdo	No lo sé
47	El implante puede quedarse debajo de la piel por 10 años.	Desacuerdo	De acuerdo	No lo sé
48	Se usa un parche cada semana, durante tres semanas, la cuarta semana no se usa parche	Desacuerdo	De acuerdo	No lo sé
49	Yo sé dónde conseguir un método anticonceptivo.	Desacuerdo	De acuerdo	No lo sé

50	Mis amigas utilizarían las pastillas anticonceptivas, inyecciones, implante, t de cobre o parche si quisieran prevenir un embarazo.	Muy en desacuerdo	Desacuerdo	No lo sé	De acuerdo	Muy de acuerdo
51	Mis amigas hablarían con sus parejas sobre métodos anticonceptivos si quisieran prevenir un embarazo.	Muy en desacuerdo	Desacuerdo	No lo sé	De acuerdo	Muy de acuerdo

52	Si quisieras utilizar las pastillas anticonceptivas, inyecciones, implante, t de cobre o parche, ¿Cuan fácil seria hacerlo?	Muy difícil	Difícil	No lo sé	Fácil	Muy fácil
53	Si quisieras hablar con tu pareja sobre anticoncepción ¿Cuan fácil sería hacerlo?	Muy difícil	Difícil	No lo sé	Fácil	Muy fácil

54	Si quisieras utilizar las pastillas anticonceptivas, inyecciones, implante, t de cobre o parche ¿Cuan segura estas de que podrías hacerlo?	Muy segura que no podría	Segura que no podría	No estoy segura	Segura que podría	Muy segura que podría
55	Si quisieras hablar con tu pareja sobre anticoncepción ¿Cuan segura estas que que podrías hacerlo?	Muy segura que no podría	Segura que no podría	No estoy segura	Segura que podría	Muy segura que podría

56	Tengo la intención de usar las pastillas anticonceptivas, t de cobre, inyecciones, implante o parche.	Muy en desacuerdo	Desacuerdo	No lo sé	De acuerdo	Muy de acuerdo
